# Hydrogen sulfide ameliorates the kidney dysfunction and damage in cisplatin-induced nephrotoxicity in rat

**Published:** 2014

**Authors:** Akram Ahangarpour, Amin Abdollahzade Fard, Mohammad Kazem Gharibnaseri, Taha Jalali, Iran Rashidi

**Affiliations:** 1 *Department of Physiology and Physiology Research Center, School of Medicine, Jundishapur University of Medical Sciences of Ahvaz, Ahvaz, Iran;*; 2 *Department of Physiology and Physiology Research Center, School of Paramedicine, Jundishapur University of Medical Sciences of Ahvaz, Ahvaz, Iran;*; 3 *Department of Pathology, Shafa Hospital, Ahvaz, Iran.*

**Keywords:** Apoptosis, Cisplatin, Hydrogen sulfide, Nephrotoxicity

## Abstract

Hydrogen Sulfide (H_2_S) prevents and treats a variety of disorders via its cytoprotective effects. However, the effects of H_2_S on rats with cisplatin (CP) nephrotoxicity are unclear. The aim was to study the effects of H_2_S on rats with CP nephrotoxicity. Thirty male Sprague-Dawley rats were divided into three groups: control group, nephrotoxic group received single dose of CP (6 mg kg^-1^) and nephrotoxic groups that received single dose 100 µmol kg^-1^ NaHS. On fifth day after injection, urine of each rat was collected over a 24-hr period. Animals were sacrificed 6 days after CP (or vehicle) treatment, and blood, urine, and kidneys were obtained, prepared for light microscopy evaluation, lipid peroxidation content and laboratory analysis. The results showed that plasma urea (226%), creatinine (271%), renal lipid peroxidation content (151%), Na and K fractional excretion, urine protein, volume and kidney weight in CP nephrotoxic rats were significantly higher and urine osmolarity and creatinine clearance lower than in controls. Increases of the proximal tubular cells apoptosis and mesangial matrix in CP nephrotoxicity group rats were observed. Hydrogen sulfide reversed the CP-induced changes in the experimental rats H_2_S prevented the progression of CP nephrotoxicity in rats possibly through its cytoprotective effects such as antioxidant properties.

## Introduction

Cisplatin (cis-diamminedichloroplatinum II, CP) is a potent anticancer drug commonly used against cancers, including testicular, cervical, head and neck malignancies. The drug is also known to produce a number of toxicities in kidney, gastrointestinal tract and nervous system.^1^^-^^3^ The drug is bio-activated to a nephrotoxicant,^4^ and it damages cell mitochondria altering the cellular transport system and eventually causes apoptosis, inflammation, necrosis and death in cells.^5^^,^^6^ The limiting factor of CP is its progressive irreversible nephrotoxicity.^7^ This has been ascribed to several mechanisms such as oxidative stress,^8^^,^^9^ modulation of nitric oxide,^10^ and modulation of adenosine.^11^

Hydrogen sulfide (H_2_S) is an endogenously produced gaseous signaling molecule with diverse physiological effects. Its production in mammalian systems has been attributed to two key enzymes in cysteine biosynthesis pathway, cystathionine β-synthase (CBS) and cystathionine γ-lyase. The rate of H_2_S production in tissue is in the range of 1 to 10 pmol sec^-1^ per mg protein, resulting in low micromolar extracellular concentrations.^12^^,^^13^ It is at these physiological concentrations that H_2_S is cytoprotective in various models of cellular injury.^14^^,^^15^ The cytoprotective effects of H_2_S are partially related to its ability to neutralize reactive oxygen species (ROS), promoting vascular smooth muscle relaxation, reducing apoptotic signaling, and reversibly modulating mitochondrial respiration.^16^ Many studies suggest exogenous and endogenous H_2_S is protective against renal ischemia-reperfusion injury. Topical administration of NaHS on kidneys plays a key role in protecting against renal ischemia and renal function can be improved.^17^^,^^18^ During ischemia, H_2_S levels drop along with kidney function.^19^ Main enzyme responsible for H_2_S production in kidney is CBS that its inhibition leads to accumulation of homocysteine and worsening renal ischemia-reperfusion injury.^20^ Another study has showed inhibition of generation of endogenous H_2_S with DL-propargylglycine an inhibitor of endogenous H_2_S formation, has improved CP-induced renal damage.^21^ But the generation of endogenous H_2_S may either limit or contribute to the degree of tissue injury caused by ischemia-reperfusion and chronic kidney disease.^18^^,^^22^ To clear up these controversies, we aimed to investigate the role of H_2_S and its potential beneficiary effects in relation to physiological, biochemical, and histological changes induced by CP in rats.

## Materials and Methods

**Animals.** Thirty male Sprague-Dawley rats weighing 180 to 220 g were purchased from animal house center of Ahvaz Jundishapur University of Medical Science, Ahvaz, Iran. All animals were housed in cages with 12/12 hr light/dark cycle at 22 ± 2 ˚C. All experimental procedures were performed according to Ahvaz University Ethical Committee for care and use of laboratory animals.

**Chemicals.** Cisplatin, other chemicals and reagents were obtained from Sigma Aldrich chemical Co. (St. Louis, USA).

**Experimental design. **Thirty rats were divided into three groups of 10 rats per each group. The groups were divided as follows; control group, untreated CP nephrotoxicity group (6 mg kg^-1^, intraperitoneally), and CP nephrotoxicity group treated with intraperitoneal NaHS (100 µmol kg^-1^, 1 hr before CP injection). The doses of NaHS were selected based on the findings from previous study that examine renal ischemia-reperfusion injury in the rat.^18^ At the 5^th^ day, rats were kept individually in metabolic cage with access to drinking water for measurement of 24 hr urinary protein and urine volume. At the end of the experimental, 10 rats from each group were sacrificed by intraperitoneal overdose of xylazine (30 mg kg^-1^, Alfasan, Woerden, The Netherlands) and ketamine (120 mg kg^-1^, Alfasan, Woerden, The Netherlands). The right kidneys were harvested stored in 10% neutral formalin and was fixed separately for light microscopy evaluation. In addition, the left kidney was dissected and rinsed with isotonic saline and then blotted dry and weighed. Then, the kidney tissue was minced and homogenized with 5% (w/v) potassium phosphate buffer (0.1 M, pH 7.4) using a homogenizer (Heidolph Silent crusher-M, Donau, Germany). Homogenate was then centrifuged at 10000 *g* for 5 min to remove nuclei and cell debris. This supernatant was used for the measurement of lipid peroxidation.

**Biochemical Analysis.** One day before the rats were killed, urine of each rat was collected over a 24 hr period and the volume was measured. All rats were killed 6 days after CP administration. Blood was collected from heart in tubes and immediately centrifuged at 3000 *g* at 5 ˚C for 10 min to separate plasma. The plasma obtained was stored frozen at –20 ˚C to await biochemical analyses. Kidneys were removed from the rats, washed with ice-cold saline, blotted with a piece of filter paper and weighed. The cortex of left kidney was excised from the medulla, and rapidly homogenized in ice-cold potassium phosphate buffer to produce 1:10 (w/v) tissue homogenate. Urine osmolality was measured by the freezing point depression method (–70 ˚C) using an osmometer (Hermann Roebling, Berlin, Germany), urine protein concentration was measured by a trichloroacetic acid (TCA) precipitation method. Trichloroacetic acid method measures the turbidity at 420 nm when a 50 µL sample is mixed with 150 μL of 30 g L^-1^ TCA at 25 ˚C.^23^ Urinary BUN, creatinine, Na and K were measured by auto analyzer (Model BT3000; Biotechnica, Rome, Italy).

**Renal histology. **After fixation of the kidneys with 10% formalin, renal tissues were sectioned and stained with periodic acid-Schiff (PAS) reagents for histological examination. Tubular damage in PAS-stained sections was examined under the microscope (×200 magnification) and scored based on the percentage of cortical tubules showing epithelial necrosis: 0 = normal; 1 = 10%; 2 = 10 to 25%; 3 = 26 to 75%; and 4 = 75%. Tubular necrosis was defined as the loss of the proximal tubular brush border, blobbing of apical membranes, tubular epithelial cell detachment from the basement membrane, or intra-luminal aggregation of cells and proteins.

**TUNEL staining. **DNA fragmentation was labeled *in situ* using an *in situ* cell death detection kit (Roche Diagnostics, Mannheim, Germany). Paraffin-embedded tissues were cut into 5 µm thick sections, and after deparaffinization and dehydration were digested with proteinase K and treated according to the protocol provided with the kit. According to TUNEL-reaction bases, labeled nucleotides were catalytically added to 3’-OH ends of DNA by terminal deoxynucleotidyl transferase in a template-independent manner. Sections were then reacted with anti-fluorescein antibody conjugated with horse radish peroxidase as a reporter enzyme. *In situ* cell death detection kit provides diaminobenzidine to produce a brown reaction product that marks the nuclei of apoptotic cells. Sections were counterstained with hematoxylin. Apoptosis positive cells were evaluated by light microscope. The number of apoptotic cells in each section was calculated by counting the number of TUNEL positive apoptotic cells in 10 × 400 fields per slide.

**Estimation of lipid peroxidation. **Lipid peroxidation in kidney tissue homogenate of all the experimental animals was determined for thiobarbituric acid reactive substances (TBARS) formation.^24^ Amount of 500 μL of supernatant was mixed with 1.5 mL of 10% trichloroacetic acid. After centrifugation (3000 *g* for 15 min), 1.5 mL of supernatant was added to 2 mL of 0.67% thiobarbituric acid and heated for 30 min at 100 ˚C. After cooling, the sample was extracted with 2 mL of n-butanol. After centrifugation at 3000 *g* for 15 min the organic phase was collected and the absorbance was read spectrophoto-metrically at 535 nm using a blank containing all the reagents except the tissue homogenate. Values were expressed as nmol g^-1^ kidney tissue. As 99% of TBARS is malondialdehyde (MDA), TBARS concentrations of the samples were calculated from a standard curve using 1, 1, 3, and 3- tetramethoxypropane.

**Statistical analysis.** Data were analyzed using one-way ANOVA and followed by Tukey’s test in SPSS (Version 16; SPSS Inc., Chicago, USA). Data are expressed as mean ± SEM. A *p* value less than 0.05 was considered as statistically significant differences.

## Results

**Effects of NaHS on kidney weight percentage. **Rats given CP lost their initial body weight, while NaHS-treated rats gained their initial body weight. In the CP nephrotoxic group, kidneys’ weight increased significantly compared to control rats (*p* < 0.01). After administration of NaHS, a significant decrease of kidney weight was found in treated CP nephrotoxic group in comparison to CP nephrotoxic group (*p* < 0.05), (Table 1).

**Effects of NaHS on plasma and urine** **biochemical analysis. **Tables 2 and 3 show the effects of treatment with saline, CP, and NaHS on concentrations of plasma urea, creatinine, Na, K and Urinalysis. Cisplatin significantly increased the concentrations of plasma urea and creatinine (*p* < 0.01). Compared with saline-treated rats, CP increased daily urine volume, while, NaHS treatments decreased urine volume (*p* < 0.01). Urine osmolality in CP-treated rats decreased compared with saline-treated controls (*p* < 0.01). In NaHS-treated rats, urine osmolality increased significantly in comparison with CP treated rats (*p* < 0.05). Urine protein excretion increased in CP-treated rats and NaHS treatment significantly decreased urine protein excretion (*p* < 0.01). 

**Table 1:** Effect of CP and treatment with NaHS on weight of body and kidneys of rats, (n = 10).

**Groups**	**Initial weight (g)**	**Final weight (g)**	**Change (%)**	**Kidneys’ weight (g)**	**Kidneys’ weight (%)**
**Controls**	221.50 ± 4.80	227.40 ± 5.10	5.90 ± 0.30	1.82 ± 0.10	0.80 ± 0.02
**CP**	201.30 ± 6.20	196.30 ± 5.80	-5.00 ± 0.50*	1.83 ± 0.10	0.93 ± 0.06*
**CP + NaHS**	216.70 ± 3.80	218.60 ± 3.80	1.90 ± 0.20*†	1.81 ± 0.20	0.82 ± 0.07†

Either Cisplatin (6 mg kg^-1^) or NaHS (100 µg kg^-1^) was given first day. Rats were weighed before any injection and in the 5^th^ day, then sacrificed.

* Values are significantly different compared to control group; *p* < 0.01).

† Values are significantly different compared to cisplatin group; *p* < 0.01).

**Table 2:** Plasma creatinine, urea, and sodium and potassium concentrations of each group, (n = 10).

**Groups**	**Creatinine** **(mg dL** ^-1^ **)**	**Urea** **(mg dL** ^-1^ **)**	**Na** **(mEq L** ^-1^ **)**	**K** **(mEq L** ^-1^ **)**
**Controls**	0.46 ± 0.03	32.10 ± 2.00	145.00 ± 1.40	4.54 ± 0.12
**CP**	1.25 ± 0.09*	72.82 ± 3.40*	143.00 ± 1.40	5.57 ± 0.13*
**CP + NaHS**	0.59 ± 0.05†	51.30 † 1.80*†	144.00 ± 0.90	5.23 ± 0.10*

* Values are significantly different compared to control group; *p* < 0.01) within the same column.

† Values are significantly different compared to cisplatin group; *p* < 0.01) within the same column.

**Effects of NaHS on kidney and tubular functions.** Table 4 shows the effects of CP and NaHS on the creatinine clearance, Na and K fractional excretion. In CP treated rats creatinine clearance decreased (*p* < 0.01) and fractional excretion of Na and K was increased (*p* < 0.01). Effects of CP on kidney and tubular functions were reversed by NaHS treatment. 

**Fig. 1:**
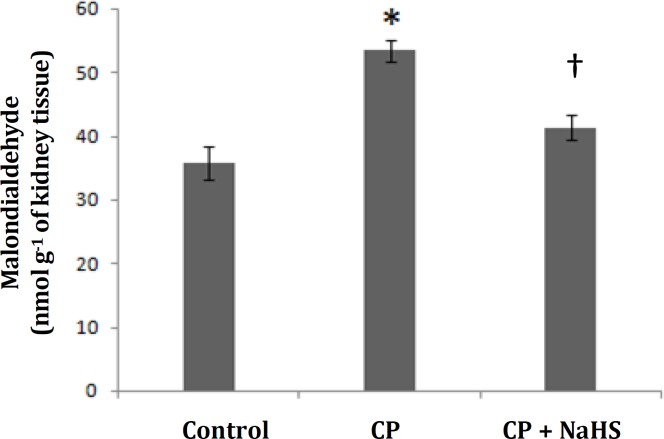
Malondialdehyde concentration as a marker for renal level of lipid peroxidation. (* indicates significant difference compared to control, and ^†^ compared to cisplatin group; *p *< 0.01), (n=10).

**Effects of NaHS on renal lipid peroxidation content. **The renal lipid peroxidation content in CP nephrotoxic rats was significantly higher compared to control rats (Fig. 1). NaHS administration resulted in a significantly lower renal lipid peroxidase content compared to the untreated CP nephrotoxic rats.

**Table 3:** Effect of CP and NaHS on urinalyses, (n = 10).

**Groups**	**Urine output (mL 24 hr)**	**Urine protein ** **(g 100 mL** ^-1^ **)**	**Urine Osmolality (osmole)**
**Controls**	8.00 ± 0.40	5.50 ± 0.20	2053.40 ± 78.00
**CP**	10.95 ± 0.30*	8.90 ± 0. 30*	1381.70 ± 76.00*
**CP + NaHS**	8.75 ± 0.50†	8.75 ± 0.50†	1691.20 ± 60.00*†

* Values are significantly different compared to control group; *p* < 0.01) within the same column.

† Values are significantly different compared to cisplatin group; *p* < 0.01) within the same column.

**Fig. 2:**
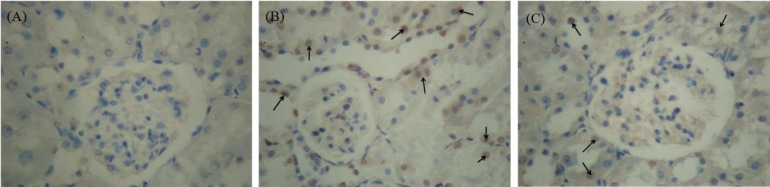
Photomicrographs of renal tissues by *in situ* cell death detection kit. NaHS attenuates the cisplatin-induced histopathological damage. (A) Control group; (B) cisplatin nephrotoxic group; (C) cisplatin nephrotoxic group treated with NaHS. Arrows show TUNEL positive cells, (TUNEL staining, 400×).

**Fig. 3:**
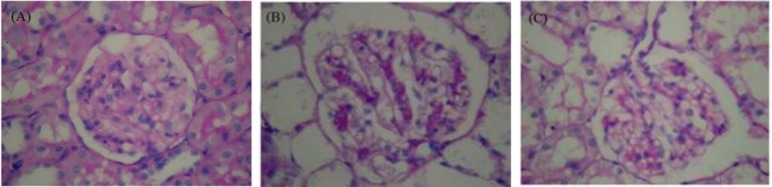
Photomicrographs of renal tissues. NaHS attenuates the cisplatin-induced histopathological damage. (A) Control group; (B) cisplatin nephrotoxic group; (C) cisplatin nephrotoxic group treated with NaHS, (PAS staining, 400×).

**Effects of NaHS on renal histology and apoptosis in CP nephrotoxic rats. **A moderate increase of mesangial matrix in the glomeruli of most CP nephrotoxic rats in comparison to the control rats has been shown in Fig. 2. This was evident by an increase in PAS-positive mesangial matrix area. Treatment with NaHS reversed the mesangial matrix accumulation caused CP. Figure 3 shows the apoptosis in the tubular epithelial cells in CP group. Apoptotic cells were not observed in tubular cells in the control rats. The apoptotic cells decreased in NaHS-treated CP group when compared to untreated CP rats.

**Table 4:** Effect of CP and NaHS on kidney and tubular functions, (n = 10).

**Groups**	**Creatinine clearance ** **(mL min** ^-1^ **)**	**creatinine clearance ** **(mL min** ^-1^ ** per gram of kidney tissue)**	**Fractional excretion of sodium **	**Fractional excretion ** **of potassium **
**Controls**	0.89 ± 0.06	0.48 ± 0.03	0.86 ± 0.03	10.32 ± 0.30
**CP**	0.47 ± 0.06*	0.25 ± 0.02*	2.69 ± 0.20*	23.92 ± 1.90*
**CP + NaHS**	0.77 ± 0.05†	0.43 ± 0.04†	1.15 ± 0.09†	12.56 ± 1.10†

* Values are significantly different compared to control group; *p* < 0.01) within the same column.

† Values are significantly different compared to cisplatin group; *p* < 0.01) within the same column.

## Discussion

Cisplatin is an effective chemotherapeutic agent for treating various solid tumors. The main limitation of this drug is nephrotoxicity.^25^^,^^26^ The reduction in body weight following CP treatment may possibly be due to the injured renal tubules, and the subsequent loss of the tubular cells to reabsorb water leading to dehydration and loss of body weight. The increased urine volume might also be the reason behind the loss of body weight. Treatment with NaHS antagonized this event significantly, but not completely. This improvement of the CP-induced body weight reduction is an evidence of the general palliative effect of NaHS on the nephrotoxicity. Further support for the salutatory action on the renal function was obtained from the improvement of the increase in plasma concentrations of creatinine and urea. The urine analysis in this work confirmed that CP caused a polyuric acute renal failure, as judged by significant increases in the volume of urine, and urine protein excretion concomitant with a significant decrease in osmolality. Treatment with NaHS reversed significantly effects induced by CP, albeit not completely. Therefore this cytoprotective and antioxidant agent could ameliorate CP nephrotoxicity. The extensive necrosis and apoptosis of the renal tissues seen in the slides of kidneys in rats treated with CP were reduced by pretreatment with NaHS. This is in line with the biochemical changes observed in serum and urine. Although the nephro-protective mechanism of NaHS is not well understood, there is evidence that NaHS has a strong antioxidant effect in several organs.^27^^,^^28^ Formation of reactive oxygen species in kidney plays an important role in CP-induced nephro-toxicity,^29^^,^^30^ and several antioxidants and thiol compounds have been shown to protect against CP nephrotoxicity.^29^^,^^31^

The present work confirms that CP raise the lipid peroxidation leaving the renal tissues vulnerable to damage by oxygen radicals responsible for tubular epithelial cell death. The present data show that pre-treatment of rats with the antioxidant agent NaHS decreased MDA as a marker for lipid peroxidation. These actions may have led to the amelioration of the histological, serum and urine biochemical indices of renal damage. Several studies have suggested that ROS may be second messengers in nuclear factor (NF-kB) activation and that antioxidants suppress NF-kB activation.^31^^,^^32^ NaHS has recently been reported to indirectly suppress NF-kB activation induced by ROS, and subsequently inhibits the transcription of a variety of inflammatory genes in renal ischemia-reperfusion Injury.^18^

The H_2_S-donor, NaHS reduces the renal injury and dysfunction caused by CP in rats. This study investigates the effects of H_2_S-donor NaHS on injury, renal function, glomerular function, and tubular function. High sodium and potassium fractional excretion indicates impaired reabsorptive function in CP-induced nephrotoxicity. In addition to the amelioration of renal dysfunction (plasma BUN and creatinine), administration of NaHS (1 hr before CP administration) has mitigated renal injury (histology), glomerular dysfunction (creatinine clearance), and tubular dysfunction (fractional excretion of sodium and potassium) caused by CP. At least two pathophysiological mechanisms lead to tubular cell death in CP nephro-toxicity.^33^^,^^34^ Necrosis is characterized by loss of membrane integrity, cellular fragmentation, and an inflammatory response. Apoptosis is characterized by cytoplasmic and nuclear shrinkage, DNA fragmentation, and breakdown of the cell into apoptotic bodies. Apoptosis is the major mechanism of early tubular cell death in acute kidney injury.^18^^,^^34^ It has been reported that H_2_S exhibit pro-inflammatory activities in endotoxic shock endotoxemia.^35^^-^37 H_2_S also has potent anti-inflammatory effects. For instance, the H_2_S donor (S-diclofenac) down-regulates the expression of inducible nitric oxide synthase, cyclooxygenase type II, cystathionine gamma-lyase (CGL), and nuclear factor *κB* in a rat model of endotoxic shock.^38^ Another H_2_S donor (NaHS) has been reported to exhibit gastro-protective effect against mucosal lesions induced by ischemia-reperfusion injury.^39^ It is known that ROS and lipid peroxidation play a role in CP induced cellular damage in kidney’s tubular epithelium^40^ Various treatments have been evaluated to reduce nephrotoxicity caused by CP.^41^^,^^42^ Histopathological examination of rat kidney was carried out to determine the number of apoptotic cells and cortical proximal tubular necrosis score. The number of apoptotic cells and proximal tubular necrosis score were statistically low in the CP + NaHS group which showed that NaHS treatment significantly reduced histopathological damage caused by CP in the kidneys.

In conclusion, we have shown that the pretreatment with NaHS could mitigate some signs of CP nephrotoxicity in rats. Further pharmacological and toxicological studies are needed to confirm NaHS safety and its efficacy against CP nephrotoxicity. It can be concluded that NaHS is a potentially nephroprotectant both in animals and humans. 
